# Bacterial Histamine as a Therapeutic Target for Abdominal Pain in Irritable Bowel Syndrome: A Literature Review

**DOI:** 10.7759/cureus.82132

**Published:** 2025-04-12

**Authors:** Hashim Mohamed Siraj, Mohammed Usaid, Saqib Shaikh, Mujahed Dalain, Yasmin Ansari, Samreen Zehra Naqvi, Raunak Hossain, Huda Anum, Catherine Andrews Ravi, Nivedita Pant, Mihir Awasarikar, Kainat Husain, Anushka Anand Hanchate, Aswija Sagam, Anand Balasubramanian

**Affiliations:** 1 Faculty of Medicine, Ivane Javakhishvili Tbilisi State University, Tbilisi, GEO; 2 Internal Medicine, Government Medical College, Latur, Latur, IND; 3 Faculty of Medicine, University of Latvia, Riga, LVA; 4 Internal Medicine, Bahria University of Medical and Dental College, Karachi, PAK; 5 Faculty of Medicine, David Tvildiani Medical University, Tbilisi, GEO; 6 Internal Medicine, Smt. Kashibai Navale Medical College & General Hospital, Pune, IND; 7 Obstetrics and Gynaecology, Jawaharlal Nehru Medical College, Aligarh, IND; 8 Faculty of Medicine, Mahatma Gandhi Mission (MGM) Medical College, Kamothe, IND; 9 Internal Medicine, Krishna Institute of Medical Sciences, Hyderabad, IND; 10 Internal Medicine, Hospital Corporation of America (HCA) Health Care Northwest, Houston, USA

**Keywords:** abdominal pain, gut microbiota, histamine, irritable bowel syndrome, visceral hyperalgesia

## Abstract

Irritable bowel syndrome (IBS) is a common functional gastrointestinal disorder characterized by abdominal pain, altered bowel habits, and discomfort. This narrative review explores the current understanding of IBS pathophysiology, diagnosis, and treatment, with a focus on the role of histamine in gastrointestinal disorders. The review summarizes the existing literature from electronic databases and manual searches. Key topics covered include the diagnostic criteria for IBS, mechanisms underlying abdominal pain, role of histamine in gastrointestinal motility, visceral hypersensitivity, and immune system dysregulation, highlighting its potential as a therapeutic target in IBS management. The narrative synthesis of findings provides insights into the complex interplay between gut microbiota, histamine production, and IBS symptomatology. Overall, this review underscores the need for further research to elucidate the mechanisms underlying IBS and histamine-related gastrointestinal disorders, with the ultimate goal of developing tailored therapeutic interventions for individuals affected by these conditions.

## Introduction and background

Irritable bowel syndrome (IBS) is a cluster of gastrointestinal disorders with unclear causes, diverse symptoms, and distinct diagnostic criteria. Its diagnosis is challenging due to symptom overlap with other chronic conditions. Globally, IBS affects about 11.2% (95% CI: 9.8%-12.8%) of people [1]. A higher incidence is seen in females and younger individuals. However, prevalence rates vary significantly across studies from 1.1% (France and Iran) to 35.5% (Mexico), with significant variability across Asia [2]. Regionalization in IBS epidemiology is necessary due to significant variations in prevalence across countries, influenced by differences in diagnostic criteria, cultural norms, and population characteristics. Focusing on regional data ensures accurate, context-specific insights and supports tailored interventions, as global prevalence estimates are often inconsistent and unreliable [2].

Quality of Life (QOL) evaluations play an essential role in assessing the impact of IBS on patients' well-being and mental health, despite its non-life-threatening nature in the majority of cases. Research indicates a notable association between IBS and a high prevalence of depression as well as compromised QOL among patients [3]. QOL is markedly diminished in females compared to males, particularly in aspects including body image perception, dysphoria, and disruptions in daily activities [4]. Among IBS symptoms, abdominal pain emerges as the most disruptive, with studies revealing a strong association between abdominal pain and adverse life impact variables and QOL, followed by intestinal gas and bloating, especially among female patients [5]. 

Histamine, a short-acting endogenous amine, exhibits widespread distribution within the human body [6]. Remarkably, 80% of IBS patients have pinpointed food, including histamine, as a potential cause of their symptoms [7]. It regulates gastrointestinal motility, gastric acid secretion, and mucosal ion transport through four histamine receptors (H1R to H4R) [6]. Activation of H1R is associated with proinflammatory responses, while H2R activation has anti-inflammatory effects. In vitro studies show that histamine can counteract the effects mediated by H2R. Pharmacological inhibition of H4R in vivo has been shown to reduce visceral hypersensitivity and decrease mast cell infiltration in the colon of germ-free mice colonized with fecal microbiome from individuals with high histamine-producing IBS [8]. Individuals with IBS have elevated levels of mast cells in the colon, often near enteric nerves, which correlates with the severity of abdominal pain. There is also a higher occurrence of histamine-producing bacteria [6,9]. The study gap in this field is largely due to the lack of data availability, particularly concerning the prevalence of histamine-producing bacterial populations in IBS patients versus healthy controls.

Targeting bacterial histamine as a therapeutic approach shows promise for addressing both IBS and histamine intolerance. By understanding histamine's role in gut function and its association with inflammation, interventions could be developed to reverse gut dysbiosis, which is often a contributing factor in IBS. Probiotics rich in strains that compete with or reduce histamine-producing bacteria, along with personalised dietary strategies, could offer a more effective treatment by targeting the root cause of IBS rather than relying solely on symptomatic treatments like histamine receptor blockers and histamine-blocking agents.

## Review

Understanding IBS

Definition and Subtypes of IBS

IBS is a long-term functional gastrointestinal disorder, distinguished by abdominal pain with defecation or altered bowel habits [10]. It can be categorized into four types according to Rome IV criteria: IBS-C (IBS with predominant constipation, characterized by hard and lumpy stools), IBS-D (IBS with predominant diarrhea, characterized by loose and watery stools), IBS-M (IBS with mixed bowel habits), and IBS-U (IBS unclassified) [11]. In some patients, IBS can occur post-infection, and so it is termed as IBS-PI (post-infectious IBS). Approximately 10% of patients with IBS believe their condition began with an infectious illness [12].

Diagnostic Criteria of IBS

Diagnosing IBS is inherently challenging due to its symptoms, such as abdominal pain, bloating, diarrhea, and constipation, that overlap with those of various gastrointestinal and systemic disorders. Therefore, clinicians must proceed cautiously, systematically excluding other potential conditions before confirming an IBS diagnosis. This process includes a thorough evaluation of the patient's symptoms to ensure they meet the Rome IV criteria of recurring abdominal pain, occurring at least once a week over the past three months, and ticking at least two of the following: I. Linked to defecation II. Altered stool frequency III. Changes in stool appearance. These criteria must persist for three months, with symptoms appearing at least six months prior to diagnosis.

A thorough history taking, including diet, medication, medical, family, surgical, and psychological factors, is essential. Assessing for warning signs in the patient, ruling out any potential concerns (like rectal bleeding) and diseases that have symptoms that mimic IBS like symptoms appearing with older age (>50 years), unexplained weight loss, congenital gastrointestinal conditions, microscopic colitis, hyperthyroidism, neurologic disease, gastrointestinal bleeding, and unexplained iron-deficiency anemia. Some specific tests that might help in differential diagnosis are complete blood cell count, C-reactive protein or fecal calprotectin test, screening for celiac disease via serologic testing, and age-appropriate screening for colorectal cancer. Lastly, diagnosing IBS into subtypes is crucial for targeted management, which can be achieved using the Bristol Stool Form Scale (BSFS) [11-13].

Mechanisms Underlying Abdominal Pain in IBS 

The mechanisms underlying abdominal pain in IBS are complex, involving a combination of physiological, biochemical, and psychological factors that together exacerbate symptoms. A deeper understanding of these mechanisms is crucial for developing more effective treatments and improving patient outcomes. Physiological mechanisms of IBS are given below. 

Abnormal Colonic Motility: Altered motility in the colon, such as abnormal contractions or changes in transit rates, is a key contributor to IBS. These abnormalities can lead to irregular bowel movements, including diarrhea or constipation, which are hallmark symptoms of IBS [14,15].

Visceral Hypersensitivity: IBS is characterized by an increased sensitivity to normal intestinal sensations, known as visceral hypersensitivity. Patients may experience exaggerated pain or discomfort from stimuli that would typically not cause pain. This heightened sensitivity is often linked to altered brain processing of gut signals, likely due to dysfunction in the gut-brain axis that amplifies or misinterprets normal sensory output [16,17].

Intestinal Inflammation and Barrier Dysfunction: Low-level inflammation, particularly in the colonic mucosa, is common in IBS patients. This inflammation can damage the intestinal epithelial barrier, increasing intestinal permeability. Disruptions to the barrier allow harmful substances, such as food antigens, bacterial products, bile acids, and serine proteases, to enter the lamina propria and bloodstream, further exacerbating symptoms. These substances can activate immune responses and stimulate afferent neurons, contributing to pain and other symptoms [16]. Tight junctions (TJs) between epithelial cells, which are essential for maintaining the gut lining, are particularly vulnerable in IBS. Inflammation, infections, or bile acid imbalances can cause these junctions to loosen, increasing paracellular permeability and worsening symptoms like bloating, pain, and diarrhea [16].

Altered Neurohormonal Activity: The neurohormonal system regulates gut function, and its dysregulation in IBS can lead to abnormal secretory mechanisms within the epithelial layer. Increased activation of the hypothalamic-pituitary-adrenal (HPA) axis, involved in the stress response, can alter gut motility and sensitivity, contributing to symptoms such as pain and bloating [16,17].

Contributing Factors and Triggers

Enhanced Brain Activity and Psychological Influences in IBS: Research indicates that individuals with IBS frequently demonstrate heightened activity in brain regions associated with pain processing and emotional regulation, such as the anterior cingulate cortex, amygdala, and dorsomedial frontal cortex. This hyperactivity may signify the interplay between stress, emotional factors, and gut function, contributing to increased pain sensitivity in IBS [17]. Psychological states like anxiety, depression, and stress significantly influence the brain-gut axis, leading to abnormal gut motility and increased visceral sensitivity. Emotional distress amplifies the perception of pain and discomfort, creating a feedback loop that exacerbates IBS symptoms. Additionally, these psychological factors alter the immune response in IBS patients. Elevated levels of proinflammatory cytokine IL-1β and reduced levels of anti-inflammatory cytokine interleukin (IL)-10 disrupt the delicate cytokine balance [15].

Intestinal Permeability: Alterations in intestinal permeability are a significant factor in IBS. Stress, food allergies, and infections can all increase permeability. For example, stress-induced release of corticotropin-releasing factor (CRF) can increase permeability. Infections, such as infectious diarrhea, can disrupt tight junctions (TJs), further increasing permeability. Certain bile acids can induce epidermal growth factor receptor phosphorylation, leading to occludin dephosphorylation and tight junction rearrangement, thereby increasing paracellular permeability. These bile acids may also affect enteric neurons, potentially increasing colonic permeability [16].

Infections and Antibiotic Use: Post-infectious IBS can develop after gastroenteritis, during which gut microbiota balance is disrupted. Antibiotic-induced dysbiosis can impair the gut’s natural defenses, contributing to intestinal barrier dysfunction and exacerbating IBS symptoms [16,17].

Dietary Triggers: Many IBS patients identify specific foods that trigger their GI symptoms, including those high in carbohydrates, fats, and biogenic amines, such as histamine-containing foods. Self-reported food intolerances are often associated with a higher symptom burden, including bloating, abdominal pain, and diarrhea, which can significantly reduce quality of life [16,18].

Current Treatment Options and Their Limitations

Care for patients with IBS is achieved by planning out an individualized, holistic approach with the main goals being dietary, lifestyle, medical, and behavioral interventions [13]. Rifaximin (antibiotic), peripheral and mixed opioid agonists, bile acid sequestrants, and antagonists of serotonin 5‑hydroxytryptamine type 3 receptors are used to treat IBS-D patients [19]. Soluble fibers (such as inulin) are shown to be effective in treating IBS-C symptoms [10]. However, the initial treatment for patients with IBS-C typically involves bulking agents and osmotic laxatives, with lubiprostone and linaclotide usually reserved for patients who find standard therapies ineffective or challenging to manage [19]. A diet low in fermentable oligosaccharides, disaccharides, monosaccharides, and polyols (FODMAPs) is found to remarkably reduce symptoms of IBS and the approach is most effective when implemented in two stages: initially, a strict avoidance of foods high in FODMAPs, followed by a gradual reintroduction based on individual symptom responses [20].

Alternative therapies include acupuncture, yoga, and herbal remedies, which some people find helpful in managing symptoms [21]. However, it's important to note that the effectiveness of these treatments can vary from person to person, and some may have limitations or side effects, as most medications may only provide temporary relief and may not address the underlying cause of IBS [19]. Additionally, dietary modifications can be restrictive and challenging to maintain, while psychological therapies may require time and commitment to see results. It's essential for individuals with IBS to work closely with their healthcare providers to find the most suitable treatment approach for their specific symptoms and needs.

Role of histamine in gastrointestinal disorders

The chemical messenger histamine is involved in many different physiological processes. It contributes to smooth muscle contraction, and vasodilation and the release of gastric acid [22]. It also plays a role in immunologically mediated inflammation, anaphylaxis, and mast cell activation syndromes [23]. Histamine influences behavioral states and opens ion channels in the neurological system via functioning as a neurotransmitter, neuromodulator, and signaling molecule [24].

Histamine Receptors in the Gastrointestinal Tract

Histamine receptors are implicated in immunological responses, gut inflammation, cancer progression, sensory signaling, and visceral pain. The H1, H2, H3, and H4 histamine receptors all have an effect on the gastrointestinal tract [25]. There various roles of these receptors in the digestive tract are summarized in Table 1.

**Table 1:** Role of histamine receptors in the gastrointestinal tract GIT: Gastrointestinal tract

Histamine H1 Receptor (H1R)	Histamine H2 Receptor (H2R)	Histamine H3 Receptor (H3R)	Histamine H4 Receptor (H4R)
Abundantly expressed throughout the gastrointestinal tract, specifically on enterocytes, myocytes, connective tissue cells, immune cells, blood vessels, and enteric nerves. They increase contractility by increasing calcium availability at the sarcoplasmic level.	Mainly present on gastric parietal cells, enterocytes, lymphocytes, myenteric ganglia, and smooth muscle cells. They facilitate cholinergic and non-cholinergic excitatory transmission in intramural neurons.	Controversial presence in the human gut.	Expressed throughout the gastrointestinal tract, liver, pancreas, and bile ducts. Implicated in immune-mediated responses in gut inflammation, gastrointestinal cancerogenesis, sensory signaling, visceral pain, and gastric ulceration. Potential target for therapy in GIT disorders as they play a crucial role in regulating various physiological and pathophysiological mechanisms in the gastrointestinal tract.

Effects of Histamine on Gut Motility and Visceral Hypersensitivity

Activation of histamine receptors (primarily H2 receptors) can stimulate smooth muscle contraction in the gastrointestinal tract resulting in increased gut motility. This affects processes such as gastric emptying and overall digestive function. For example, nizatidine, an H2 receptor antagonist, has been shown to alleviate symptoms of GERD and improve functional dyspepsia by promoting gastric emptying. 

Histamine receptors, particularly the H1 and H2 receptors, have been implicated in the modulation of visceral hypersensitivity. Activation of histamine H1 receptors has been associated with the induction of visceral hypersensitivity in various animal models. On the other hand, histamine H2 receptors have been suggested to play a role in modulating visceral sensitivity and pain perception in the gastrointestinal tract. The complex interplay between histamine receptors and visceral hypersensitivity highlights their potential as therapeutic targets for conditions characterized by heightened visceral sensitivity [26].

Histamine receptors play a significant role in intestinal inflammation [25]. The effects of histamine receptors in intestinal inflammation are summarized in Table 2.

**Table 2:** Role of histamine receptors in intestinal inflammation

Effects by	Histamine H1 Receptor (H1R)	Histamine H2 Receptor (H2R)	Histamine H3 Receptor (H3R)	Histamine H4 Receptor (H4R)
Receptor Agonists	Activation of these receptors can lead to increased vascular permeability, smooth muscle contraction, and pro-inflammatory cytokine release, contributing to inflammation.	Stimulation of H2 receptors can increase gastric acid output, exacerbating inflammation in situations such as gastritis or ulcers.	The role of H3 receptors in intestinal inflammation is less obvious, however, their presence in the gastrointestinal tract indicates a potential modulatory role in neurotransmitter release and immunological responses.	H4 receptors are involved in immune-mediated responses to gut inflammation. Stimulation of H4 receptors can lead to immune cell recruitment, cytokine release, and modulation of inflammatory responses in the gut.
Receptor Antagonists	H1 receptor antagonists have been shown to reduce inflammation in experimental colitis models.	H2 receptor antagonists are clinically utilized to lower gastric acid release and treat diseases characterized by excessive acid production.	-	H4 receptor antagonists decrease experimental colitis in animal models, indicating a possible therapeutic target for inflammatory bowel illnesses.

IBS patients have dysregulated expression of histamine receptors, particularly the H2 receptor, and a decreased percentage of circulating H2R+ monocytes. This imbalance is linked to reduced anti-inflammatory signaling as H2R typically suppresses proinflammatory cytokine responses to bacterial ligands via Toll-like receptors (TLRs) [27]. Additionally, inflamed gastrointestinal mucosa shows increased expression of H1R, H2R, and H4R, with H2R correlating with tumor necrosis factor (TNF)-α expression and H4R with interferon (IFN)-γ expression, suggesting their involvement in mucosal inflammation. An altered balance between H2R (regulatory) and H4R (proinflammatory) may further contribute to immune dysregulation [27]. On the other hand, a possible correlation between H2R antagonist (H2RA) use and IBS risk indicates a considerably greater risk of IBS among those who used H2RAs compared to non-users [28]. 

The ion channel transient receptor potential vanilloid 1 (TRPV1), which is mainly expressed in sensory neurons, has been demonstrated to be sensitized by histamine. It is essential for the detection and control of many stimuli, especially those pertaining to temperature and pain. It accomplishes this by activating H1, which causes dorsal root ganglia (DRG) neurons to respond more strongly to calcium. It is believed that this sensitization plays a role in the visceral hypersensitivity that patients with IBS experience. When compared to a placebo, the H1 antagonist ebastine significantly increased the percentage of IBS patients who experienced significant symptom improvement after 12 weeks of treatment, according to a proof-of-concept clinical trial. This implies that inhibiting H1 could provide relief from IBS symptoms. According to the findings, mast cell mediators, specifically histamine, are essential for sensitizing TRPV1, which may cause IBS patients to perceive more visceral pain. Experiments utilizing rectal biopsy samples from both IBS patients and healthy participants provided evidence for this [29]. 

The pathophysiology of colitis, a disorder that has certain symptoms with IBS, has been linked to the H4R, according to research conducted on animal models. For instance, it has been demonstrated that in models of severe colitis, the absence of H4R expression exacerbates symptoms, suggesting that H4R may play a protective function in gut inflammation. It is hypothesised that targeting H4R, which is implicated in inflammatory processes, could offer therapeutic advantages for controlling the symptoms of IBS and other inflammatory gastrointestinal disorders [30]. These findings suggest a complex relationship between histamine dysregulation and IBS, warranting further investigation.

Evidence Linking Histamine Dysregulation to IBS Symptoms

Histamine dysregulation has been implicated in the pathogenesis of irritable bowel syndrome (IBS) and its associated symptoms. In a study by Wang (2007), it was found that histamine levels were increased in the terminal ileum of IBS-D patients compared to controls [31]. Mucosal biopsies from the terminal ileum, duodenum, and jejunum were collected via endoscopy and analyzed using immunohistochemistry to quantify enterochromaffin (EC) cells and mast cells, marked by serotonin (5-HT) and tryptase antibodies, respectively. 5-HT levels in small intestine samples were measured using high-performance liquid chromatography-electrochemistry (HPLC-ECD) after perchloric acid homogenization. ANOVA was used to assess differences in cell counts and serotonin levels across patient groups. These methods enabled the assessment of EC and mast cell distribution and density, along with 5-HT concentrations in the small intestinal mucosa of IBS patients compared to healthy controls, which provided valuable insight into the role of serotonin and immune cells in modulating gut sensitivity and motility, offering a foundation for more targeted and effective IBS therapies [31].

Furthermore, mast cells, which are known to release histamine among other mediators, have been implicated in the pathogenesis of IBS. According to a study by Krammer et al. (2019), which examined 36 case-control studies, IBS patients had significantly higher mucosal mast cell counts or densities (30 out of 36) than healthy controls [32]. This implies that mast cell numbers and symptoms related to IBS are positively correlated, especially between symptoms like bloating and stomach pain and the mast cell count. It has been reported that mast cell mediators including histamine and tryptase activate and intensify neural signaling, which leads to visceral hypersensitivity, a symptom of IBS. This is validated by Findings that link the production of mast cell tryptase to levels of protease-activated receptor (PAR)-2, which is linked to symptoms like stomach pain. Additionally, it noted that IBS patients had higher levels of degranulating mast cells, suggesting that mast cells are not only more prevalent but also actively participate in the inflammatory processes linked to IBS. The data points to mast cells' role in the pathophysiology of IBS, indicating that they may elicit low-grade inflammation and aid in the emergence of IBS symptoms [32]. Overall, the dysregulation of histamine and mast cells in the gut mucosa may play a role in the development and manifestation of IBS symptoms, highlighting the potential importance of immune system dysfunction in this functional bowel disorder [33].

Bacterial histamine production in the gut

Overview of Histamine-Producing Bacteria in the Gut

Endogenously produced histamine and its receptors are extensively studied, whereas exogenous histamine production is mostly associated with food-borne illnesses like dairy and fish poisoning. Mou et al. conducted a comprehensive search for histamine-producing bacterial species using the Genome Taxonomy Database (GTDB) and Unified Human Gastrointestinal Genome database (UHGG), encompassing 31,910 and 4,644 genomes, respectively [34]. They identified 117 histamine-producing species, with 57 using pyruvoyl-dependent histidine carboxylase and 39 using pyridoxal-5’-phosphate (PLP) dependent histidine carboxylase. Pleisomonas shigelloides was found to contain both pathways. Species using the PLP-dependent histidine decarboxylase (HDC) were limited to just the Proteobacteria phylum, whereas the pyruvoyl dependent HDC containing species were from the following six phyla: Verrucomicrobiota, Proteobacteria, Fusobacteriota, Firmicutes, Bacteroidota, Actinobacteriota. In the gut flora, some of the common genera are Bacteroides, Clostridium, Bifidobacterium, Fusobacterium, and Lactobacillus [34]. 

Interestingly, 21 of the 97 putative histamine-producing bacterial species identified from GDTB are from a clade where greater than half of the strains lack the operon enabling them to produce and/or secrete histamine. This strain specificity makes it a bigger challenge as identifying species alone may not tell us enough information about their histamine production, and molecular typing for specific strains may be needed for further specificity. This finding becomes exceedingly pertinent when it comes to conducting trials that compare IBS gut microflora to healthy controls and may lead to faulty or incomplete conclusions [34]. 

Some of the well-known producing genera and species that frequently recur in literature are Proteus, Staphylococcus, many under Enterobacteriaceae, numerous in the Actinobacteriota and Firmicutes phyla, species including *Clostridium perfringens*, *Klebsiella aerogenes* (formerly *Enterobacter aerogenes*), *Proteus mirabilis*, *Enterococcus faecalis* [9,35,36]. *Morganella morganii* in particular was found to be associated with the severity of asthma symptoms in asthmatic patients probably owing to its high histamine-producing capacity [9,37]. Prominent histamine-producing species and their corresponding HDC pathways are summarized in Table 3.

**Table 3:** Prominent histamine producing species and their corresponding histidine decarboxylase pathways *Strain specific

Histamine Producing Species	Histidine Decarboxylase Pathway
Klebsiella aerogenes	Pyridoxal-5'-Phosphate (PLP)-dependent
Clostridium perfringens	Pyruvoyl-dependent
Lactobacillus reuteri*	Pyruvoyl-dependent
Pseudomonas aeruginosa	Pyridoxal-5'-Phosphate (PLP)-dependent
Morganella morganii	Pyridoxal-5'-Phosphate (PLP)-dependent
Pleisomonas shigelloides	PLP-Dependent and Pyruvoyl-Dependent

A limitation of this study that may be proposed is the variability in testing methodology as the identification of histidine decarboxylase operons, performed using the hmmscan obtained results for both PLP and pyruvoyl-dependent histidine decarboxylases, however the 3D modelling structure analysis for histidine-histamine antiporter(hdcP) and the PLP-dependent histidine decarboxylase is unavailable and hence leaves room for further testing and potential variability in data [34].

Factors Influencing Bacterial Histamine Production

Factors influencing bacterial histamine production primarily include underlying diseases, conditions, and dietary habits [9,37]. Sánchez-Pérez et al. discussed a dietary treatment for histamine Intolerance (HIT), caused by a deficiency in diamine oxidase (DAO), the enzyme responsible for metabolizing histamine. DAO deficiency leads to excess histamine absorption, triggering gastrointestinal and extra-intestinal symptoms due to the widespread distribution of histamine receptors [35]. DAO deficiency caused by histamine intolerance was found to be associated with the changed population of gut microbiota, as the group with HIT, compared to the healthy group, had significantly lower proportions of healthy gut bacteria and an abundance of histamine-producing bacteria leading to histamine-induced dysbiosis. A restrictive diet for five histamine-intolerant patients over nine months, consisting of low histamine content and supplemental DAO, significantly lowered the relative abundance of 21 bacterial families (below 1%) and reduced the total number of bacterial families from 79 to 58 [9].

Barcik et al. conducted an analysis of fecal samples of 161 volunteers and found significantly elevated levels of histidine decarboxylase (HDC) gene in adult patients with asthma. A more striking finding in the same study is that non-obese adult asthmatic patients had the highest levels of HDC genes in fecal samples compared to their obese counterparts. This gives us the idea that histamine sources for dysbiosis and non-GI conditions are interlinked [37].

Relationship Between Gut Dysbiosis and Histamine Production

While the general link between gut microbiota and gastrointestinal discomfort has been known, the pathophysiological mechanisms behind it are not well-researched. De Palma et al. investigated this by collecting stool microbiome samples from five IBS patients with low histamine in urine (IBSLH) and transplanting them into 34 germ-free mice [8]. The mice showed increased intestinal sensitivity to mechanical stimuli evaluated by measuring action potential discharge in single afferent colonic nerves. In the next phase, 119 germ-free mice underwent fecal microbiome transplantation from an IBS patient with high urine histamine (IBSHH), an IBS patient with low urine histamine (IBSLH), and a healthy control (HC). Mice transplanted with samples from the IBS patient with high urine histamine experienced hyperalgesia compared to those with samples from the IBSLH and HC.

Furthermore, Ross et al. tested minimal media inoculated stool samples from 23 IBS patients and three HC donors. From the isolated colonies of IBS patients, 61% produced histamine compared to only 33% in HC, and 20% of the IBS colonies degraded histamine while only 11% did so in the HC colonies [38]. The two previously cited sources along with the DAO deficiency caused dysbiosis in Sánchez-Pérez et al. give us ample evidence to draw the significant relation between histamine and gut dysbiosis.

Therapeutic Potential of Targeting Bacterial Histamine in IBS

A study conducted by De Palma et al. examined gut microbial histamine production by incubating cecal contents from human microbiota-transplanted mice with excess histidine [7]. Mice colonized with microbiota from an IBS patient with high urinary histamine produced 38 times more histamine than those colonized with microbiota from an IBS patient with low urinary histamine or a healthy control. A low-fermentable carbohydrate diet reduced histamine production in high-histamine microbiota mice but had no effect on low-histamine or healthy microbiota mice. Bacterial histamine moderately correlated with visceral mechanosensitivity. These findings were validated with stool samples from IBS patients, finding higher histamine production in samples from high-histamine patients. In an independent cohort, microbiota from high-pain periods produced more histamine than from low-pain periods.

*Klebsiella aerogenes* was identified as the main histamine producer in high histamine patients, while Enterococcus species were dominant in low histamine patients. *K. aerogenes* was more abundant in high-histamine patients and decreased with a low fermentable carbohydrate diet. Another cohort confirmed higher K. aerogenes prevalence in IBS patients compared to controls [8]. Comparative genomic analysis showed *K. oxytoca* lacked the HDC gene, and germ-free mice colonized with *K. aerogenes* had significantly higher histamine levels than those with *K. oxytoca*. Histamine production peaked at pH 7.0 and was influenced by lactic acid levels, which varied between high and low histamine microbiota [8].

Bacterial histamine affected the intestinal neuroimmune system, increasing dorsal root ganglion neuron excitability, which was inhibited by H1 and protease-activated receptor 2 (PAR2) antagonists. High histamine microbiota increased colonic mast cells and their proximity to neurons. A *K. aerogenes* strain without the HDC gene had lower mast cell numbers and histamine levels, while engineered *E. coli* with the HDC gene increased both. Histamine-induced mast cell migration via H4R signaling, with increased H4R expression in high-histamine microbiota mice. Blocking H4R reduced visceral pain and mast cell numbers, suggesting H4R mediates bacterial histamine effects on the gut neuroimmune system [8].

These findings suggest that gas production and bowel distension are not the primary causes of pain in IBS patients on high fermentable diets. This might explain why only some IBS patients benefit from a low fermentable diet [39,40]. It's crucial to identify patients who will benefit from this diet, as restricting dietary fiber can further reduce microbial diversity in Western populations. The research also indicates that identifying bacteria like K. aerogenes, which produce histamine in the gut, could lead to tailored therapies. These might include microbiota-directed therapies, H4 receptor antagonists, or specific dietary interventions for IBS patients with chronic abdominal pain [8].

## Conclusions

Managing abdominal pain in IBS remains challenging due to the limited and often temporary effectiveness of current treatments. Emerging evidence suggest that histamine dysregulation and mast cell activation play a significant role in IBS pathophysiology, pointing to a potential link with immune system dysfunction. Studies indicate that IBS patients have a higher prevalence of histamine-producing gut bacteria, which may contribute to visceral hypersensitivity. Notably, blocking histamine, particularly H4R, has shown promise in symptom reduction. These findings highlight the need for further research in microbiota-targeted therapies, including H4R antagonists, fecal transplantation, and bacteriophage therapy, to develop more personalized and effective treatment strategies for IBS.
